# Minimally invasive presacral approach for revision of an Axial Lumbar Interbody Fusion rod due to fall-related lumbosacral instability: a case report

**DOI:** 10.1186/1752-1947-5-488

**Published:** 2011-09-29

**Authors:** Anders Cohen, Larry E Miller, Jon E Block

**Affiliations:** 1 The Brooklyn Hospital Center, 121 Dekalb Avenue, Brooklyn, NY 11201, USA; 2 Miller Scientific Consulting, Inc., 422 Mountain Wasp Drive, Biltmore Lake, NC 28715, USA; 3 Jon E. Block, PhD, Inc., 2210 Jackson Street, Suite 401, San Francisco, CA 94115, USA

## Abstract

**Introduction:**

The purpose of this study was to describe procedural details of a minimally invasive presacral approach for revision of an L5-S1 Axial Lumbar Interbody Fusion rod.

**Case presentation:**

A 70-year-old Caucasian man presented to our facility with marked thoracolumbar scoliosis, osteoarthritic changes characterized by high-grade osteophytes, and significant intervertebral disc collapse and calcification. Our patient required crutches during ambulation and reported intractable axial and radicular pain. Multi-level reconstruction of L1-4 was accomplished with extreme lateral interbody fusion, although focal lumbosacral symptoms persisted due to disc space collapse at L5-S1.

Lumbosacral interbody distraction and stabilization was achieved four weeks later with the Axial Lumbar Interbody Fusion System (TranS1 Inc., Wilmington, NC, USA) and rod implantation via an axial presacral approach.

Despite symptom resolution following this procedure, our patient suffered a fall six weeks postoperatively with direct sacral impaction resulting in symptom recurrence and loss of L5-S1 distraction. Following seven months of unsuccessful conservative care, a revision of the Axial Lumbar Interbody Fusion rod was performed that utilized the same presacral approach and used a larger diameter implant. Minimal adhesions were encountered upon presacral re-entry. A precise operative trajectory to the base of the previously implanted rod was achieved using fluoroscopic guidance. Surgical removal of the implant was successful with minimal bone resection required. A larger diameter Axial Lumbar Interbody Fusion rod was then implanted and joint distraction was re-established. The radicular symptoms resolved following revision surgery and our patient was ambulating without assistance on post-operative day one. No adverse events were reported.

**Conclusions:**

The Axial Lumbar Interbody Fusion distraction rod may be revised and replaced with a larger diameter rod using the same presacral approach.

## Introduction

Lumbar fusion surgery is performed on over 100,000 patients per year in the US [1]. Open lumbar fusion procedures have inherent procedural risks, regardless of the surgical approach. Posterior and transforaminal approaches may result in significant soft tissue injury while an anterior approach jeopardizes critical organs and major vessels. Minimally invasive spinal surgery techniques offer the spine surgeon an alternative that yields comparable clinical and radiographic results with less iatrogenic soft tissue injury and minimal blood loss.

The AxiaLIF (Axial Lumbar Interbody Fusion) System (TranS1, Inc., Wilmington, NC) utilizes a small paracoccygeal incision for percutaneous access to the presacral area for discectomy and fusion of L5-S1, which is a markedly different anatomical access route compared to other surgical approaches.

The AxiaLIF System results in similar fusion rates compared to other fusion procedures, but with less risk of nerve injury since the access route avoids critical neurovascular and musculoligamentous structures [2]. Although complications with the AxiaLIF procedure are uncommon [3], revision surgery may be necessary in cases of pseudarthrosis or recalcitrant postoperative pain. DeVine and colleagues describe the removal of an AxiaLIF implant via a paramedian retroperitoneal approach [4]. The purpose of this case report was to describe what is, to the best of our knowledge, the first account of an AxiaLIF revision procedure via a presacral approach.

## Case presentation

**Figure 1:**
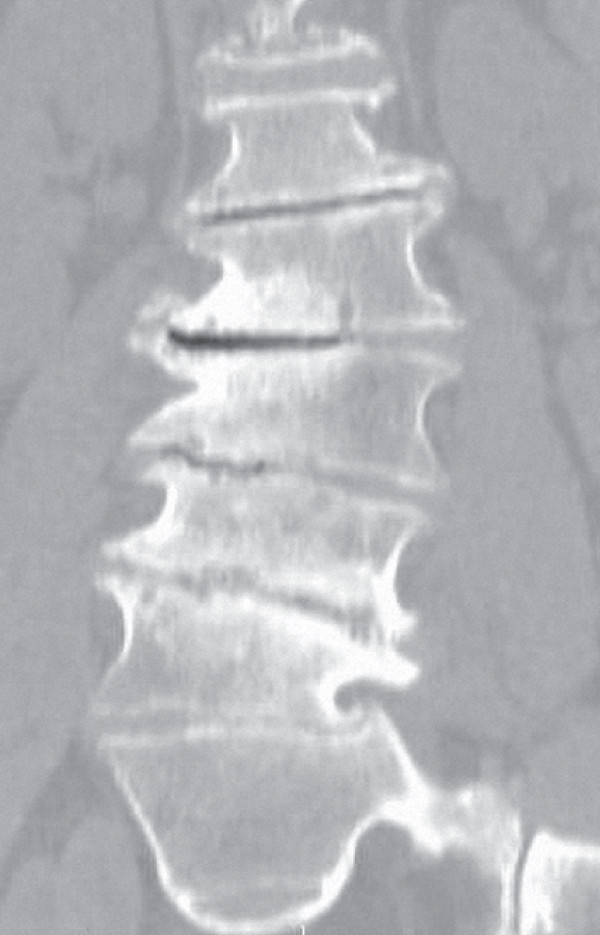
**Pre-operative coronal computed tomography (CT) image of our patient, a 70-year-old man with marked thoracolumbar scoliosis, degenerative osteoarthritic changes characterized by high-grade osteophytes, endplate sclerosis and significant inter-vertebral disc space collapse**.

Our patient, a 70-year-old Caucasian man, presented to our facility with marked thoracolumbar scoliosis, osteoarthritic changes characterized by high-grade osteophytes, and significant intervertebral disc collapse and calcification (Figure 1). Our patient required crutches during ambulation and reported intractable axial and radicular pain.

**Figure 2:**
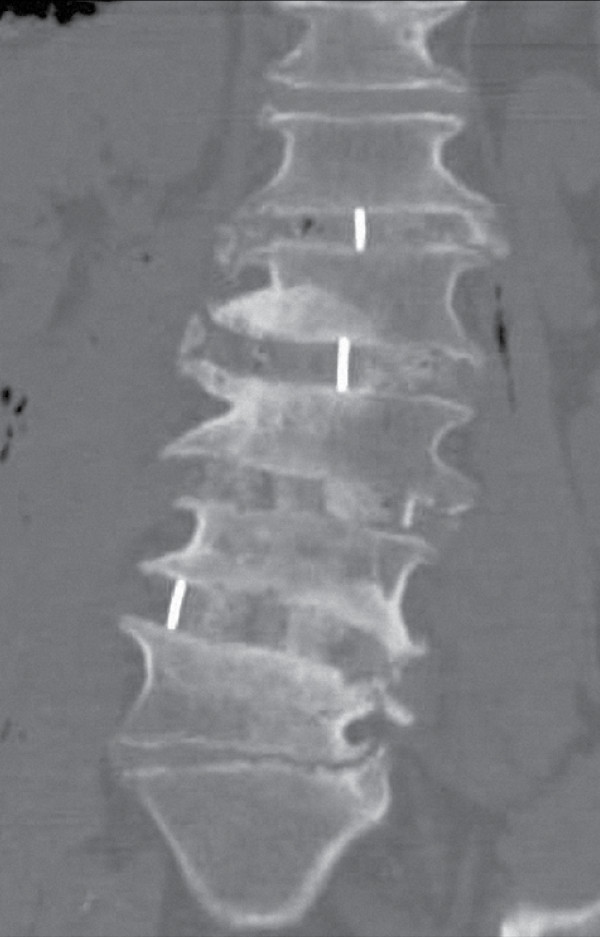
**Post-operative coronal computed tomography (CT) image of the index operative intervention involving an 'eXtreme Lateral Interbody Fusion' (XLIF) procedure**. Multi-level L1-L4 surgical reconstruction and instrumentation. Focal lumbosacral symptoms persisted due to L5-S1 disc space collapse.

**Figure 3:**
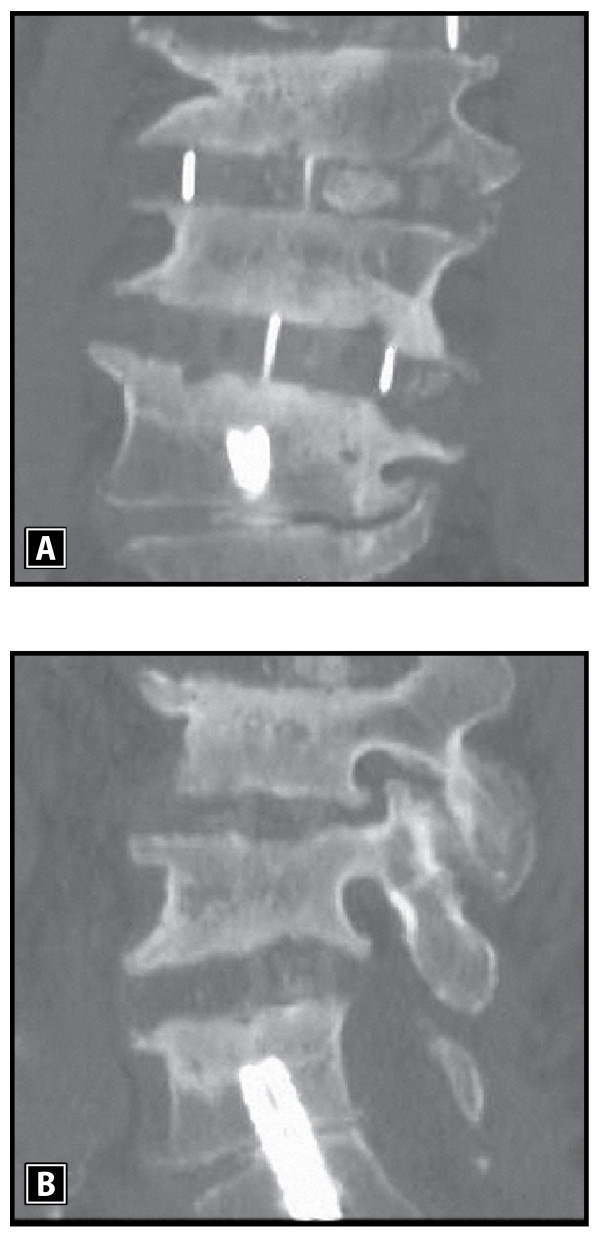
**Coronal (A) and sagittal (B) computed tomography (CT) images demonstrating improved inter-vertebral L5-S1 disc space distraction and stabilization following minimally invasive AxiaLIF rod implantation with commensurate symptom resolution and functional improvement**.

**Figure 4:**
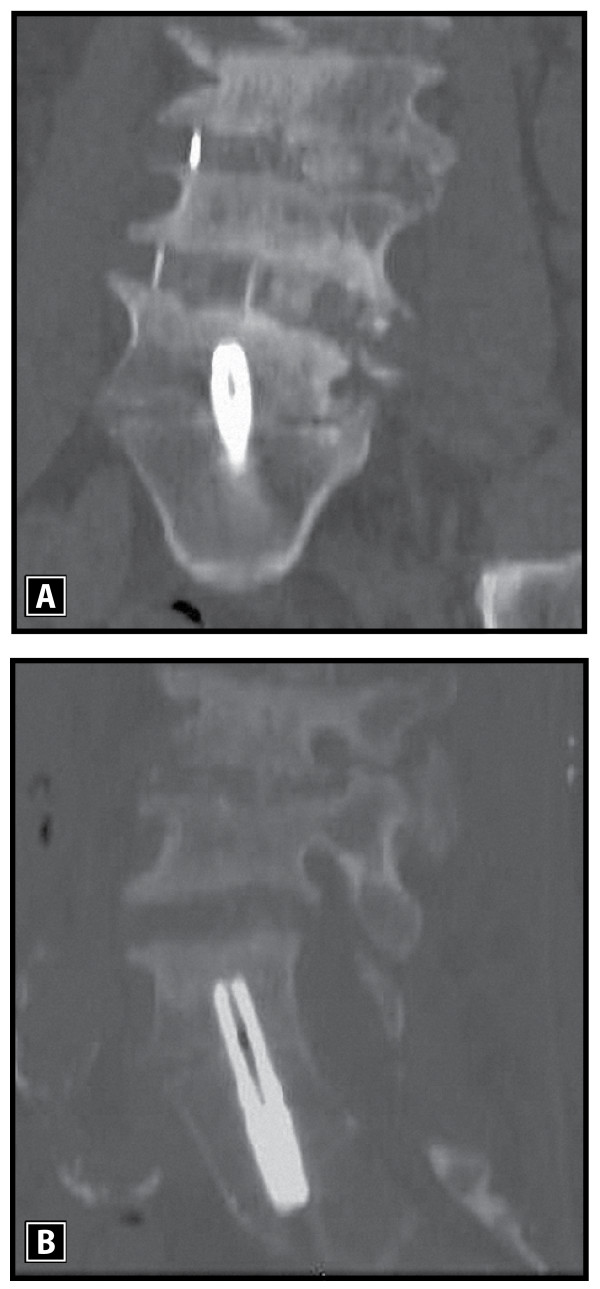
**Coronal (A) and sagittal (B) computed tomography (CT) images demonstrating loss of L5-S1 disc space with recurrence of symptoms due to direct sacral impaction following a fall six weeks after the index surgery**.

Multi-level reconstruction of L1-4 was accomplished with extreme lateral interbody fusion (Figure 2). However, focal lumbosacral symptoms persisted due to disc space collapse at L5-S1. Lumbosacral interbody distraction and stabilization was achieved four weeks later with the AxiaLIF System and rod implantation via an axial presacral approach as previously described (Figure 3) [5]. Although our patient's symptoms resolved following this procedure, he suffered a fall six weeks postoperatively with direct sacral impaction that resulted in radicular symptom recurrence and loss of L5-S1 distraction (Figure 4). Our patient unsuccessfully attempted conservative treatment measures for seven months after the fall and, ultimately, underwent revision of the AxiaLIF rod, which utilized the same presacral approach but used a larger diameter implant.

**Figure 5:**
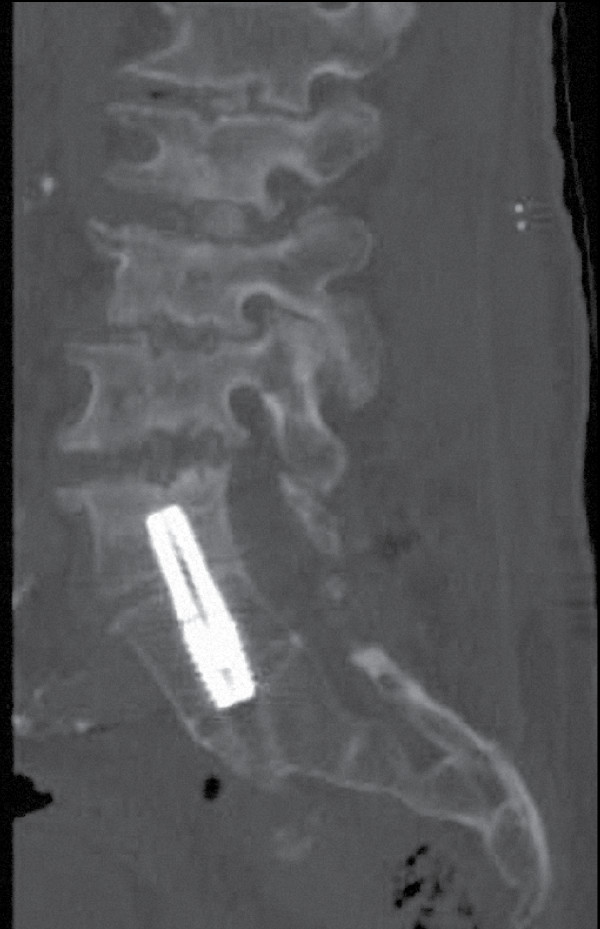
**Sagittal computed tomography (CT) image following successful revision and replacement of the AxiaLIF rod**. Note re-establishment of L5-S1 disc space distraction with improvement of radicular symptoms.

Minimal adhesions were encountered upon presacral re-entry. A precise operative trajectory to the base of the previously implanted rod was achieved using fluoroscopic guidance. A rod driver was advanced over a guidewire to the sacrum and the implant was rotated counter-clockwise for a full revolution. Next, a reverse-threaded extraction tool was advanced, engaged onto the implant, and rotated in a counter-clockwise direction until the AxiaLIF rod was freed. Surgical removal of the implant was successful with minimal bone resection required. Next, the L5-S1 intervertebral space was packed with allograft mixed with bone marrow aspirate. A larger diameter AxiaLIF rod of the same length was then implanted to attain secure fixation and joint distraction was re-established (Figure 5).

His radicular symptoms resolved following revision surgery and our patient was ambulating without assistance on postoperative day one. No adverse events were reported.

## Discussion

This case demonstrated that the AxiaLIF interbody distraction rod may be revised and replaced with a larger diameter rod using the same minimally invasive presacral approach. Revision surgeries are quite common following lumbar fusion surgery as up to 20% of patients require reoperation within 10 years [6]. Patients who undergo revision surgery following lumbar fusion experience more procedure-related complications than those undergoing an index fusion [7]. It is, therefore, paramount not only to refine existing surgical techniques for the index procedure, but also to ensure the safety and effectiveness of revision procedures.

The advantage of the presacral access route utilized during the AxiaLIF revision was that it greatly minimizes the risk of severe iatrogenic injury versus open revision procedures due to the lack of critical anatomical structures in the presacral corridor. To date, only 40 single-level and 10 two-level AxiaLIF rods have been reported as explanted out of over 8000 implants [8]. To the best of our knowledge, this report is the only experience with a minimally invasive AxiaLIF revision and demonstrates the feasibility of this procedure. The revision technique described herein should be performed by spine surgeons experienced with the AxiaLIF system, since the risk for procedural complications may be greater in the presence of a scarred presacral tract.

Despite the infrequency of reported AxiaLIF failures, spine surgeons must understand the indications for revision and alternative treatment methods when revision is not feasible. Indications for an AxiaLIF revision are identical to those for other spinal fusion techniques such as infection, pseudoarthrosis, and implant loosening or migration. Although the technique described herein represents a minimally invasive treatment option for patients who require re-intervention, isolated cases of rod removal by an anterior surgical approach have been reported. Alternatively, the rod can be left in place and supplemental techniques can be utilized to achieve fixation including delivery of additional graft material to the disc space or implantation of additional instrumentation such as plates and pedicle screws.

## Conclusions

The AxiaLIF interbody distraction rod may be revised and replaced with a larger diameter rod using the same presacral approach. Additional experience is needed to confirm the feasibility of this procedure in a larger group of patients.

## Consent

Written informed consent was obtained from the patient for publication of this case report and any accompanying images. A copy of the written consent is available for review by the Editor-in-Chief of this journal.

## Competing interests

The authors declare that they have no competing interests.

## Authors' contributions

AC performed the surgery and collected data from our patient. AC, LM, and JB interpreted the data from our patient and were involved in drafting the manuscript. All authors read and approved the final manuscript
